# Comparison of the Results of Therapy for cT1 Renal Carcinoma with Nephron-Sparing Surgery (NSS) vs. Percutaneous Thermal Ablation (TA)

**DOI:** 10.3390/jpm12030495

**Published:** 2022-03-18

**Authors:** Michał Rusinek, Marek Salagierski, Waldemar Różański, Bartłomiej Jakóbczyk, Michał Markowski, Marek Lipiński, Jacek Wilkosz

**Affiliations:** IInd Department of Urology, Medical University of Lodz, al. Kościuszki 4, 90-419 Łódź, Poland; michal_rusinek@wp.pl (M.R.); urologia@kopernik.lodz.pl (M.S.); waldemar.rozanski@office365.umed.pl (W.R.); bjakobczyk@poczta.onet.pl (B.J.); miklipa@poczta.onet.pl (M.L.); j.wilkosz@kopernik.lodz.pl (J.W.)

**Keywords:** renal cancer, nephron sparing surgery, thermoablation, complications

## Abstract

Implementation of ultrasonography (USG), computed tomography (CT) and magnetic resonance imaging (MRI) into abdominal cavity diagnostics enabled early detection of cT1 graded renal cancers. According to European Association of Urology (EAU) and Polish urological Association (PUA) recommended method of treatment is sparing resection of renal parenchyma with tumour—nephron-sparing surgery (NSS). In selected cases other methods such as thermal ablation (TA) or cryoablation can be introduced /1/. Objectives: To evaluate the results of treatment of cT1 renal tumours with the use of NSS and TA methods. Material and methods: 140 patients with cT1 renal carcinoma were treated in 2nd Department of Urology of Medical University of Lodz between 2014 and 2017. Neuron-sparing surgery was performed in 56 cases (40%), while percutane-ous thermal ablation (TA) in 84 cases (60%). Demographic data, clinical data (lab results, Charlson index), nephrometry data (tumour size, location, R.E.N.A.L. score) post-operative data (Clavien-Dindo classifica-tion) were investigated. Histopathology results, Fuhrman malignancy grading, as total three-year survival of patients were evaluated. The following methods were used for statistical evaluation: Chi2, Fisher, W Shapiro-Wilk, U Mann-Whitney tests, Kaplan-Meier’s curve and Cox model. The results were displayed in a form of median and upper and lower quartile values (25–75%). Results: No statistical differences in gender nor left/right kidney location were observed. Patients, who underwent TA were at average 10 years older and had multiple comorbidities (median age for TA was 79, for NSS 68; median Charlson index for TA was 5 and for NSS was 3). TA patients had lesser haematological values (Hb, Ht). R.E.N.A.L. scoring demonstrated comparable nephrometry in both groups. NSS procedure was open laparotomy without temporary clamping of renal vessels. Surgical margins of resected tumours were negative. TA was performed with Cool-Tip Covidienequipment with the use of Cluster electrode and was ultraso-nography-guided. Post-treatment complications evaluated with the use of Clavien-Dindo classification were slightly more frequent for NSS method. Patients after NSS were discharged at average after 8.5 days and after TA after 3 days. Histopathological type and Fuhrman malignancy grading were comparable in both groups. TA treated patients’ death risk was 9-fold of that observed in NSS treated patients. There was 1 death for each group in perioperative period. Conclusion: 1. NSS was associated with slightly higher side effect rate but resulted in prolonged survival. 2. TA was applied to elderly patients with comorbidities. Despite less invasive treatment this group had poorer/reduced survival. 3. Charlson Comorbidity Index (CCI) and the treatment method were relevant survival factors in patients treated due to cT1 renal cancer tumours.

## 1. Objectives

To evaluate the results of treatment for cT1 renal tumours with the use of NSS and TA.

## 2. Introduction

In 2018, according to the GLOBOCAN registry, 403,362 new kidney cancer cases were recorded worldwide [1]. This accounts for 3% of all cancers in adults and is ranked 12th among all neoplasms (9th in men and 14th in women) [2,3]. More than 59% of kidney cancers are diagnosed in developed countries, mostly in Europe, North America and Australia, with fewer tumors diagnosed in Africa, India and China. Kidney cancer is most frequently diagnosed in the Czech Republic, Slovakia and Lithuania, as far as Europe is concerned [4].

Kidney cancer occurs mainly in men (75%) aged of 60 and older [5]. The root cause of this neoplasm is not precisely determined. Cancer risk factors include chemical factors, with smoking being the leading factor. The risk of developing renal cell carcinoma among smokers is greater by 54% among men, and 22% among women [6].

The introduction of ultrasonography (USG), computed tomography (CT) and magnetic resonance imaging (MRI), into abdominal cavity diagnostics, enabled early detection of asymptomatic types of renal cancers. The resection of tumours or kidneys with tumours became the standard renal tumour treatment method.

The research on the optimal method of treatment for small asymptomatic renal tumours is pending [7,8,9,10]. Current guidelines for the tumour’s diameter of up to 7 cm recommend maximum sparing of renal parenchyma (NSS) [11].

Nephron loss influence on cardiovascular diseases is observed [12]. Patients with numerous comorbidities, disabling regular surgery for anaesthesiological or general reasons, are investigated. Experimental methods include thermal ablation, cryoablation, brachytherapy or stereotactic radiotherapy. Stereotactic radiotherapy is a unique method of treating pathological lesions, which consists of administering one or more large doses of radiation to the tumour area, with a minimal exposure of surrounding tissues. Stereotactic radiosurgery is used as part of radical, as well as palliative and analgesic, treatment. In many cases, it is a reasonable alternative to a riskier classical surgical treatment [13,14,15,16]. In this paper, we compare the outcome/results of treating cT1 renal cancer with 4 cm diameter in patients, with the use of NSS techniques and ultrasound-guided TA.

## 3. Material

Retrospective analysis of 140 patients with T1N0M0 renal carcinoma treated in 2nd Department of Urology of Medical University of Lodz between 2014 and 2017 was performed. The analysis/investigation was approved by the Ethics Committee of Medical University of Lodz. Nephron-sparing surgery was used in 56 cases, while percutaneous high radio frequency thermal ablation (RFA) was used in 84 cases.

## 4. Methods

Renal tumours were diagnosed with the use of ultrasonography, computed tomography and magnetic resonance imaging. Histopathology examination of removed tumour was performed afterwards. In patients treated with TA, ultrasonography-guided biopsy was performed. Comorbidities were evaluated with Charlson Comorbidity Index. Nephrometry parameters were evaluated with R.E.N.A.L. score. Post-treatment complications were evaluated with the use of Clavien-Dindo classification [17,18,19]. Patients with small T1 renal tumours with peripheral or intermediate location per R.E.N.A.L. score were qualified to NSS, which was open laparotomy (ONSS) without clamping renal vessels. Patients with small T1 renal tumours with peripheral or intermediate location per R.E.N.A.L. score but who had objections related to age or general conditions, had renal contraindications (single kidney, tumours in both kidneys) or who had not consented to surgery were qualified to thermal ablation.

Thermal ablation was performed with monopolar Cool-tip RF ablation system (Covidien, Mansfield, MA, USA) [20,21]. Urea, creatinine and potassium levels were examined before and after operation.

Imaging examinations such as ultrasound, CT, MRI, PET/CT were performed 3, 6 and 12 months after ablation followed by CT and MRI 6-monthly for the next 2 years. Patient follow-up was completed in 2020. Patients’ death data were obtained from the Ministry of Digitalisation after obtaining respective approvals.

Statistical analysis was performed with the use of statistical packet STATISTICA 13.1 licensed by Medical University of Lodz. Nominal variables were displayed as a count of observations and percentage values calculated for investigated and control groups. Chi^2^ test was used for comparison. For low count of observations to increase conservatism of the test, the Fisher’s exact test was used. Continuous variables due to non-normal distribution pattern (verified with Shapiro-Wilk test) were displayed in a form of median and upper- and lower-quartile values (25–75%). Both groups were compared with the use of Mann-Whitney U test. For survival analysis Kaplan-Meier’s curve and single regression Cox proportional hazards model were applied. To eliminate the bias caused by clinical variables which substantially varied between investigated and control groups, multiple regression Cox proportional hazards model was used considering only those variables which were significant or almost significant (*p* < 0.1) in single regression model. The results of model application were presented as regression factor, odds ratio with 95% confidence interval (95% CI) and *p* value.

Clinical data of treated patients are presented in Table 1 and Table 2. Table 1 contains nominal variables and Table 2 continuous variables. In the group of 140 patients with T1a renal tumour there were 86 (61.43%) men and 54 (38%) women. Median age was 67.5 years (59–74.5). Among 140 patients, only 1 patient did not have comorbidities; others had between one and three comorbidities and median value in Charlson comorbidity index was 2 (0–4). Lab values for analysed group were within normal ranges. We found that 76 (54.29%) patients had tumour located in left kidney and 64 (45.71) in right kidney. Median diameter of tumour was 28 mm (23–34.5 mm). Further, 126 (90%) tumours were classified as cT1a and 14 (10%) as cT1b.

In R.E.N.A.L. nephrometry score the location of tumour was exophytic in 128 cases (91.43) or intermediate in 12 cases (8.57%), which proves the right selection of patients to the applied treatment. As such, 56 patients (40%) underwent partial resection of parenchyma containing tumour and in 84 patients (60%) thermal ablation was performed; 133 patients (95%) had no complications, 3 patients (2.14) had minor complications and 2 patients (1.43%) major ones. Two patients (1.43) died during perioperative period.

**Figure 1:**
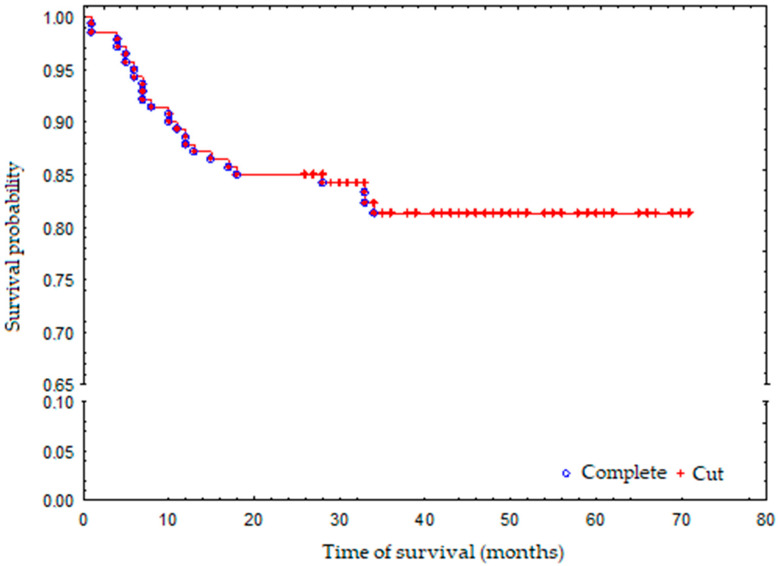
Survival probability in the whole group.

The average duration of hospitalisation was 8 days. In 123 cases clear cell carcinoma was confirmed in histopathology. Other cases were papillary type I (6 cases (4.29%)), papillary type II (8 cases (5.71%)) and chromophobe renal carcinoma (3 cases (2.14%)) respectively. Fuhrman nuclear grade value (malignancy) was 1 to 2 in 123 cases (87.86). During 3-year follow-up, 25 patients died in both groups. Probability of survival of both groups was 81% (Figure 1). Initial clinical features of both patient groups (NSS and TA) were split into nominal variables and are presented in Table 1, and continuous variables presented in Table 2.

## 5. Results Overview and Discussion

Surgical treatment is the standard way of managing renal carcinoma. The type of procedure depends on the size and the location of the tumour, as well as the coexistence of plugs, made of cancer cells, in the renal vein and vena cava inferior. Patients disqualified from surgical treatment can be treated with various forms of tumour ablation and stereotactic radiotherapy [13,14,15]. Ablation of pT1 tumours is safe and associated with low incidence of complications. Efficacy and survival period after NSS remain controversial [22]. Patients treated in our centre formed a homogenous group, as far as TMF grading is concerned (T1N0M0), and the same lab assays and imaging examinations were performed. The median age of the group was 67, which is the age when renal tumours occur most frequently [23]. The group treated with partial resection (NSS) was 10 years younger and the median age of this group was 68. The median age of the group treated with thermal ablation (TA) was 79. In the NSS group, only three patients lived to 75 years of age, while in the TA group, there were 32 such (38.09%) patients. These correspond with the observations of other authors [24,25]. In the treated group, renal cancer occurred more often in men than in women (2:1), and we did not observe relevant differences in frequency between left and right kidney. The presence of comorbidities expressed in the Charlson comorbidity index enabled the assessment of the treatment. The average index value for the whole group was 2 points, while for the NSS group, 3 points, and for the TA group, 5 points, respectively. According to Benegas M. P. et al., patients with Charlson index >1, who were treated with NSS but not nephrectomy, have a greater chance of maintaining better glomerular filtration by 2.5-fold, smaller risk of development of chronic kidney insufficiency, as well as occurrence of cardiovascular events [25]. The American Urological Association (AUA) recommends thermal ablation with the following comorbidities: diabetes, hypertension, chronic renal disease, cerebrovascular and cardiovascular diseases and high surgical and anaesthesiologic risk [26]. In our material, probably due to the elderly age of patients treated with the use of TA, the Charlson index is higher than in other authors’ publications [13]. Lab assays performed prior to the procedure were all within normal ranges (Table 2). Red cells and haemoglobin levels were statistically higher in patients treated with NSS, compared to those treated with TA (Table 2). This indicates a better general condition of patients undergoing open surgery. Platelet levels were comparable in both groups. Nephrometry analysis showed treated tumours were comparable in size and location in the left or right kidney. However, tumour diameters in patients treated with TA were significantly larger than in patients treated with NSS. The R.E.N.A.L. scoring system was implemented to unify the anatomical classification of tumours [27,28,29,30,31,32]. This system is used for the evaluation of NSS and TA treatment. The system describes tumour size and its exophytic or endophytic placement, as well as distance to the renal pelvis and sinus and location within kidney. The use of the system enabled the selection of comparable groups, nephrometry wise (1% of tumours were of exophytic and 8% of intermediate placement). NSS was performed as open laparotomy and patients qualified to this treatment method had to meet ASA classification criteria values = 1 or 2. The tumours in all operated patients had negative resection margin. Ficarra V. et al., in their paper, published in 2018, confirmed that positive resection margin was observed in 6.7% of resected tumours [33]. NSS patients were hospitalised significantly longer compared to TA patients. No post-treatment complications were observed in 95% of cases. Minor post-treatment complications were observed in three patients who had undergone NSS. Severe post-treatment complications were observed in one patient, who had undergone NSS, and in one patient who had undergone TA. Perioperative mortality was comparable in both groups. One NSS patient died due to bleeding and cardiovascular complications and one TA patient died due to thermal intestine injury, followed by peritonitis. No differences were observed in histopathological type and Fuhrman malignancy grading in either group. There were no deaths due to kidney cancer in either group of patients (treated with NSS and thermoablation) for 36 months, despite the differences in the nephrometry assessment of the R.E.N.A.L score in both groups.

**Figure 2:**
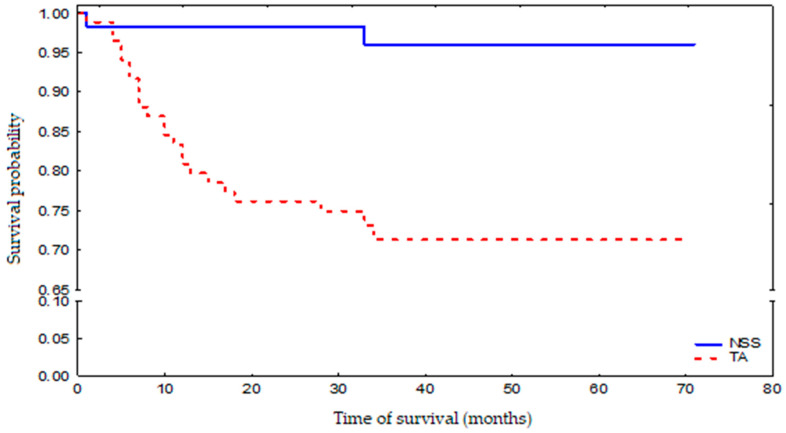
Survival probability in NSS-treated and TA-treated patient groups.

During the follow-up period, 25 patients died: 2 NSS patients (3.5%) and 23 TA patients (27.3%), which is highly statistically significant. Three-year survival probability for the whole group was 81% (Figure 1). The death risk in the TA-treated group was 9-fold higher than that observed in the NSS-treated group, which was mostly associated with a greater number of additional illnesses and more advanced age of the patients who have undergone TA (Figure 2). The observed overall survival of patients in both groups did not depend on the neoplastic disease, but on the age of the patients (patients treated with renal-saving surgery were at average 10 years younger than patients treated with thermoablation) and their comorbidity-associated general condition (patients treated with thermoablation had statistically significantly more comorbidities than those treated with renal-sparing surgery, which was reflected in higher Charlson Comorbidity Index).

## 6. Conclusions

1. NSS was associated with a slightly higher side effect rate but resulted in prolonged survival;
2. TA was applied to elderly patients with comorbidities. Despite less invasive treatment, this group had poorer survival;
3. The aim of presenting two widely different methods for small kidney tumour treatment was to demonstrate their application for various age groups and clinical conditions.

## Figures and Tables

**Table 1:** Comparison of distribution of nominal variables in the whole group and between NSS-treated and TA-treated group.

Variable	Investigated Group (*n* = 140)	NSS (*n* = 56)	TA (*n* = 84)	*p* Value
**sex**				
Men	86 (61.43%)	37 (66.07%)	49 (58.33%)	0.3568
Women	54 (38.57%)	19 (33.93%)	35 (41.67%)	
**Kidney**				
Left	76 (54.29%)	28 (50%)	48 (57.14%)	0.4056
Right	64 (45.71%)	28 (50%)	36 (42.86%)	
**R.E.N.A.L.**				
4	88 (62.86%)	42 (75%)	46 (54.76%)	0.0387
5	28 (20%)	6 (10.71%)	22 (26.19%)	
6	12 (8.57%)	3 (5.36%)	9 (10.71%)	
7	6 (4.29%)	2 (3.57%)	4 (4.76%)	
8	5 (3.57%)	2 (3.57%)	3 (3.57%)	
9	1 (0.71%)	1 (1.79%)	0 (0%)	
**CLAVIEN-DIDNO**				
1	133 (95%)	51 (91.07%)	82 (97.62%)	0.0906
2	3 (2.14%)	3 (5.36%)	0 (0.00%)	
3	2 (1.43%)	1 (1.79%)	1 (1.19%)	
5	2 (1.43%)	1 (1.79%)	1 (1.19%)	
**cT1 feature**				
cT1a	126 (90%)	51 (91.07%)	75 (89.29%)	0.9541
cT1b	14 (10%)	5 (8.93%)	9 (10.71%)	
**Type of cancer**				
clear cell	123 (87.86%)	44 (78.57%)	79 (94.05%)	
papillary type 1	6 (4.29%)	4 (7.14%)	2 (2.38%)	
papillary type 2	8 (5.71%)	6 (10.71%)	2 (2.38%)	0.0534
chromophobe	3 (2.14%)	2 (3.58%)	1 (1.19%)	
**Grading**				
G1–G2	123 (87.86%)	50 (89.28%)	73 (86.90%)	0.6726
G3	17 (12.14%)	6 (10.71%)	11 (13.10%)	
**Deaths**				
All in follow-up period	25 (17.8%)	2 (3.5%)	23 (27.3%)	0.0005
Perioperative	2 (1.43%)	1 (1.75%)	1 (1.19%)	1.0000

**Table 2:** Comparison of continuous variables between NSS-treated and TA-treated groups.

	Total (*n* = 140)	NSS (*n* = 56)	TA (*n* = 84)	*p* Value
**Variable**	Mediana (25–75%)	Mediana (25–75%)	Mediana (25–75%)	*p* value
Age	67.5 (59–74.5)	68 (11.24–63.50)	79 (10.95–72.00)	<0.0001
CHARLSON [score]	2 (0–4)	3.00 (1.86–1.00)	5 (2.28–3.00)	0.0002
Tumour diameter [mm]	28 (23–34.5)	30 (8.62–24.50)	35.50 (7.48–31.00)	<0.0001
Erytrocyt/RBC [mln/mL]	4.58 (4.17–4.94)	5 (0.68–4.80)	4.92 (0.72–4.43)	0.0032
RDW-CV [%]	13,6 (12.85–14.6)	13,90 (1.20–13.10)	14.75 (1.91–13.90)	<0.0001
RDW-SD [%]	44.5 (41.85–47.2)	44.55 (3.60–42.55)	48.3 (5.89–45.85)	<0.0001
Haemoglobin [g/L]	13.7 (12.5–14.85)	15 (1.61–14.20)	14.65 (2.00–13.20)	0.0074
Platelets [1000/uL]	231 (197.5–281.5)	291.50 (68.20–237.50)	272.50 (61.83–224.50)	0.0744
Neutrophiles [1000/uL]	4.7 (3.75–5.95)	5.93 (2.08–4.76)	6.02 (2.56–4.69)	0.8951
Lymphocytes [1000/uL]	1.81 (1.53–2.16)	2.21 (0.64–1.78)	2.15 (0.78–1.84)	0.6490
Monocytes [1000/uL]	0.64 (0.5–0.78)	0.76 (0.27–0.62)	0.81 (0.27–0.66)	0.2605
PLR	130.44 (98.4–161.58)	174.04 (63.94–147.25)	159 (51.85–120.3)	0.0959
NLR	2.59 (1.84–3.31)	4.17 (1.88–2.47)	3.2 (1.47–2.64)	0.939
LMR	3.07 (2.16–3.85)	4.18 (1.49–3.25)	3.56 (1.17–3.02)	0.3300
Days in the hospital	8 (3.07–11.0)	8.1 (1.24–8.00)	3.00 (2.30–3.00)	<0.0001
